# Subclinical Ventricular Dysfunction in Long-Term Acromegaly Assessed by Speckle-Tracking Echocardiography

**DOI:** 10.3389/fendo.2022.812964

**Published:** 2022-02-04

**Authors:** Patricia Gadelha, Eduardo C. L. Santos, Jose Castillo, Lucio Vilar

**Affiliations:** ^1^ Division of Endocrinology, Hospital das Clínicas, Pernambuco Federal University, Recife, Brazil; ^2^ Division of Echocardiography, Hospital das Clínicas, Pernambuco Federal University, Recife, Brazil; ^3^ Divison of Echocardiography, Escola de Ecocardiografia de Pernambuco, Recife, Brazil

**Keywords:** acromegaly, left ventricular strain, echocardiography, left ventricular dysfunction, speckle-tracking echocardiography

## Abstract

**Introduction:**

Symptomatic heart disease may be present in patients with advanced-stage acromegaly. However, earlier assessment of subclinical ventricular systolic dysfunction can be accomplished through speckle-tracking echocardiography (STE) for the study of myocardial strain. The few such studies in this population to date have produced conflicting results. This study was performed to evaluate the parameters of ventricular strain in patients with acromegaly with no cardiac symptoms.

**Methods:**

In this prospective observational study, STE was performed in patients with active acromegaly with no detectable heart disease and in a control group to assess ventricular dysfunction through global longitudinal strain (GLS), radial strain, circumferential strain, and twist. The left ventricular (LV) ejection fraction, LV mass index, and relative wall thickness were also compared between the groups.

**Results:**

Twenty-five patients with active acromegaly (median age, 49 years; median disease duration, 11 years) and 44 controls were included. LV hypertrophy was more prevalent in the acromegaly group (40% vs. 19%, p < 0.01). The LV ejection fraction was similar between the groups (65.2% ± 5.99% vs. 62.9% ± 7.41%). The mean GLS (−18.8 ± 2.49 vs. −19.7 ± 3.29, p = 0.24), circumferential strain (−16.7 ± 3.18 vs. −16.6 ± 3.42, p = 0.90), and twist (14.6 ± 5.02 vs. 15.1 ± 3.94, p = 0.60) were not significantly different between the groups.

**Conclusion:**

Despite showing higher rates of LV hypertrophy, patients with long-term acromegaly had no impairment of ventricular contractility as assessed by strain echocardiography when compared with a control group.

## Introduction

Acromegaly is a rare disease characterized by growth hormone (GH) and insulin-like growth factor type 1 (IGF-1) hypersecretion, and it is usually caused by a GH-producing pituitary adenoma (1). New effective treatments for acromegaly that have been developed in the last several years have improved the survival of patients with acromegaly (2, 3). Nonetheless, cardiovascular disease is still the most prevalent comorbidity and an important cause of mortality in patients with acromegaly (4, 5), thus requiring early diagnosis and appropriate treatment.

Acromegaly can cause a typical cardiomyopathy characterized by concentric biventricular hypertrophy (mainly of the left ventricle) that is associated with impaired diastolic function, which can progress to systolic heart failure (5). However, most echocardiographic studies have shown that systolic function is preserved and that symptomatic heart failure is an uncommon and late complication (6, 7). Therefore, identifying patients with acromegaly who have increased cardiovascular risk is the main challenge in avoiding this late phase of acromegalic cardiomyopathy.

Standard two-dimensional echocardiography has traditionally been applied to analyze structural and functional heart abnormalities in patients with acromegaly. Recent studies have employed more modern techniques such as speckle-tracking echocardiography (STE), which has allowed for the assessment of myocardial deformation or strain. This is a well-validated, reproducible tool that offers a more sensitive study of myocardial contractility (8). Strain describes deformation of the myocardium that occurs during the cardiac cycle in the longitudinal, circumferential, and radial planes (9). STE can be used to evaluate the global longitudinal strain (GLS), which reflects left ventricular (LV) function and may help to identify subclinical LV systolic dysfunction. Additionally, STE can be used to study other components of ventricular contraction, such as radial strain, circumferential strain, and ventricular rotation (twist) (10). Twist is the result of counterclockwise rotation of the LV apex with simultaneous clockwise rotation of the base. Reductions in GLS portend worse cardiovascular outcomes (11), and evaluation of such reductions has been applied to the early detection of heart valve diseases, myocardial ischemia, hypertrophic cardiomyopathy, and cancer therapy-related cardiac dysfunction as well as in the preclinical detection of cardiac involvement in systemic diseases, adding important information regarding the cardiovascular prognosis (12, 13).

Only a few studies have examined the effects of active acromegaly on ventricular deformation parameters, and these studies have shown conflicting results (14, 15). In the present study, we analyzed whether patients who had long-term active acromegaly with no cardiovascular symptoms would exhibit impaired parameters of ventricular deformation (strain) compared with a matched control group according to the results of STE. Such analysis could serve as a tool to better identify subclinical LV dysfunction in patients with acromegaly.

## Patients and Methods

### Study Design and Patients

The study group comprised consecutive patients with active acromegaly who were recruited from the Neuroendocrinology Clinic of Hospital das Clínicas, Universidade Federal de Pernambuco, Recife, Brazil from July 2019 to June 2021. This was a cross-sectional, single-center study.

Acromegaly was diagnosed based on typical clinical features associated with elevated serum IGF-1 levels for age and sex, lack of GH suppression to <1 ng/mL during an oral glucose tolerance test, and positive pituitary magnetic resonance imaging findings (16, 17). Acromegaly was considered uncontrolled if the serum IGF-1 level was higher [>1.2 times the upper limit of normal (ULN)] than a standard cut-off age-adjusted normal range (18). The presumed duration of acromegaly was assessed by comparing old photographs and conducting interviews. The inclusion criteria were an age of >18 years and the presence of acromegaly not cured by neurosurgery.

The control group comprised 44 age- and sex-matched healthy individuals. Patients with a history of coronary artery disease, heart failure, valvular heart disease, stroke, peripheral arterial disease, chronic renal failure, and pregnancy were excluded from both groups.

Our study was approved by the human research ethics committee of our institution and complied with the Declaration of Helsinki. All patients and healthy volunteers provided informed consent.

### Clinical and Biochemical Parameters

Obesity was defined as a body mass index of ≥30 kg/m^2^. Arterial hypertension was defined as a systolic blood pressure of ≥140 mmHg and/or diastolic blood pressure of ≥90 mmHg or the treatment of previously diagnosed arterial hypertension (19).

Plasma glucose, glycosylated hemoglobin, total cholesterol, high-density lipoprotein cholesterol, and triglycerides were measured in the morning after a 12-hour fasting period. Low-density lipoprotein cholesterol was calculated by the Friedewald formula. Diabetes mellitus was diagnosed according to the American Diabetes Association criteria (20).

Plasma GH and IGF-1 levels were measured with a chemiluminescence assay kit (IMMULITE 2000; Los Angeles, CA, USA). The IGF-1 level was expressed as how many times it was above the ULN.

### Standard Echocardiography

All healthy volunteers and patients with acromegaly underwent a complete two-dimensional transthoracic echocardiographic study as recommended by the American Society of Echocardiography (21) using a phased-array ultrasound system (Vivid i; GE Healthcare, Chicago, IL, USA). All studies were performed by a single investigator (E.C.L.S.).

In all cases, the LV dimensions, volumes, and ejection fraction and the left atrial dimensions were measured, and complete two-dimensional Doppler and tissue Doppler studies were performed. Ventricular volumes were calculated using the apical four- and two-chamber views, and the LV ejection fraction was calculated using the biplane Simpson formula. The LV mass (LVM) was calculated using the linear method as recommended by the American Society of Echocardiography for cardiac chamber quantification by echocardiography in adults (21). The relative wall thickness (RWT) was calculated from the LV end-diastolic dimension and posterior wall thickness with the following formula: RWT = (2 × posterior wall thickness)/LV end-diastolic dimension. The LVM was indexed (LVMi) to the body surface area to determine LV hypertrophy. LV hypertrophy was defined as an LVMi of >115 g/m^2^ in men or >95 g/m^2^ in women. An increase in the LVMi was classified as having concentric geometry (RWT > 0.42) or eccentric geometry (RWT ≤ 0.42). Concentric remodeling was diagnosed if the LVMi was normal but the RWT was >0.42 (21).

### Speckle-Tracking Echocardiography

The images selected for strain analysis were stored digitally for later offline analysis using dedicated software (EchoPAC PC, workstation version 204; GE Healthcare) performed by another investigator (J.C.) who was blinded to the patients’ clinical data.

LV GLS was assessed in the four-, two-, and three-chamber apical views using automated function imaging software (10). The quality of myocardial tracking was visually assessed with the possibility of manual adjustments. The LV walls were divided into six segments in each apical view, and the tracking quality and strain value were assessed for each LV segment. The mean global longitudinal peak systolic strain was calculated for each view. GLS was the average of the value that was obtained for three apical views. By convention, GLS is a negative percentage number, indicating fiber shortening or myocardial thinning. Greater degrees of deformation therefore translate to numerically lower strain values. To avoid any misunderstanding, current guidelines recommend presenting the numerical data or referring to the change in deformation; thus, a reduced absolute strain value reflects contractile dysfunction (10, 21–24).

Short-axis views of the left ventricle at the basal, mid, and apical levels are necessary to obtain circumferential and radial strain, whereas the basal and apical views are needed to measure twist. Radial strain draws positive curves, and circumferential strain draws negative curves. Twisting is generated by opposite rotation of the LV base and apex. Basal and apical LV rotation are expressed in degrees, and twist is the result of the difference between apical and basal rotation (22).

### Statistical Analysis

Statistical analyses were performed using R software (25). For descriptive analyses, categorical variables are presented as percentage and frequency and numerical variables are presented as mean ± standard deviation when normally distributed; they are presented as median when non-normally distributed. The Shapiro–Wilk test was used to test normality. The chi-square test or Fisher’s exact test was used to compare categorical variables. Student’s t test or the Wilcoxon non-parametric was used to compare numerical variables. Correlations between numerical variables were analyzed using Pearson’s or Spearman’s correlation test according to their distributions. A p value of <0.05 was considered statistically significant.

## Results

### Clinical and Biochemical Characteristics of Study Population

In total, 34 patients with acromegaly were enrolled in the study, and 9 were excluded (1 with prior acute myocardial infarction, 1 with mitral regurgitation, and 7 with cured acromegaly). The mean age of the 25 remaining patients with active acromegaly was 49.9 ± 13.2 years, and 64% of the patients were female. The mean IGF-1 level was 1.68 times (range, 1.04–2.54) the ULN. The median disease duration was 11 years (range, 9–18 years). In the acromegaly group (n = 25), 13 patients (52%) had arterial hypertension and 16 patients (64%) had diabetes mellitus. Twenty patients (80%) had a history of transsphenoidal surgery. To achieve postsurgical hormonal control, 18 patients (72%) were using somatostatin analogs as monotherapy or in combination with cabergoline. Among the remaining seven patients not using somatostatin analogs, five had a recent diagnosis of acromegaly and were awaiting surgery. Only seven patients (28%) had controlled disease, defined as an IGF-1 level of up to 1.2 times the ULN (18).

The control group comprised 44 patients [28 women (63%); mean age, 48.4 ± 8.03 years]. Among the 44 patients, 23 (52%) had hypertension and 11 (25%) had diabetes mellitus.

In the acromegaly group, 71% of patients with arterial hypertension were on ACE-I/ARB (angiotensin converting enzyme inhibitors or angiotensin receptors blockers) therapy, the same occurring in 67% of those in the control group. This difference was not statistically significant.

The main clinical and biochemical characteristics of both groups are presented in **Table 1**.

**Table 1:** Clinical and biochemical features of patients with acromegaly vs. control group.

	Acromegaly Group	Control Group	p
	n = 25	n = 44	
Sex (female/male, n)	16 / 9	28 / 16	1
Age (years)	49.9 ± 13.2	48.4 ± 8.03	0.605
Body mass index (kg/m^2^)	30.7	27.9	0.062
Diabetes mellitus (%)	16 (64%)	11 (25%)	0.003
Arterial hypertension (%)	13 (52%)	23 (52%)	1
Disease duration (years)	11 (9-18)	NA	
IGF-1 levels (ULN)	1.68 (1.04-2.54)	NA	
Controlled disease (%)	7 (28%)	NA	

Values are presented as the mean ± SD. ULN, upper limit of normal; NA, not applicable.

### Standard Echocardiographic Evaluation of LV Function

The LV ejection fraction was normal and similar between the groups (65.16% ± 5.99% vs. 62.9% ± 7.41%, p=0.19). Patients in the acromegaly group showed larger systolic volumes (31.8 ± 4.78 vs. 28.2 ± 3.81 mL, p = 0.001) and diastolic volumes (49.1 ± 5.96 vs. 45.0 ± 4.45 mL, p = 0.002) than subjects in the control group. The mean LVMi was higher in the acromegaly group than control group (87.9 ± 27 vs. 69.3 ± 17.5, p = 0.001). Compared with the control group, the acromegaly group presented more LV hypertrophy: 10 patients (40%) had changes in LV morphology and 7 (28%) had LV hypertrophy, whereas in the control group, 82% presented with normal geometry and only 1 (2%) had LV hypertrophy (p = 0.013). The findings of conventional echocardiography are shown in **Table 2**.

**Table 2:** Standard echocardiography parameters in patients with acromegaly vs. control group.

	Acromegaly Group	Control Group	p
	n = 25	n = 44	
LVEF Simpson (%)	65.2 ± 5.99	62.9 ± 7.41	0.195
LVEDV (ml)	49.1 ± 5.96	45 ± 4.45	0.002
LVESV (ml)	31.8 ± 4.78	28.2 ± 3.81	0.001
LVMi (g/m^2^)	87.9 ± 27	69.3 ± 17.5	0.001
RWT	0.39 ± 0.08	0.38 ± 0.08	0.487
Normal LV geometry (%)	15 (60)	36 (81.82)	0.013
LV concentric hypertrophy (%)	4 (16)	1 (2.27)	
LV eccentric hypertrophy (%)	3 (12)	0 (0)	
LV concentric remodeling (%)	3 (12)	7 (15.91)	

LVEF, left ventricular ejection fraction; LVEDV, left ventricular end diastolic volume; LVESV, left ventricular end systolic volume; LVMi, left ventricular mass index; RWT, relative wall thickness.

### LV Myocardial Strain Assessed by STE

**Figure 1:**
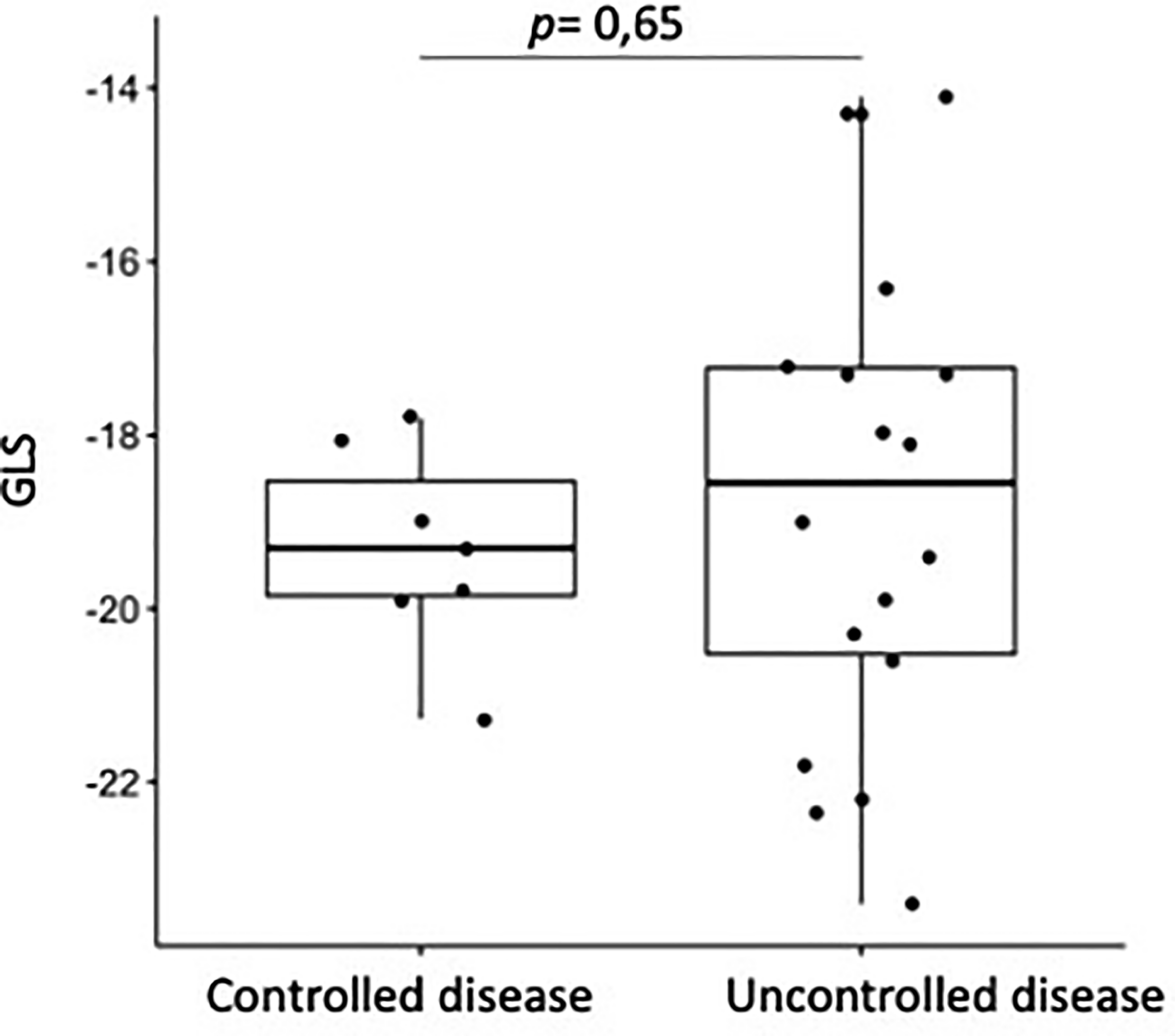
Correlation between GLS values and disease control.

The mean GLS values were not significantly different between the groups (−18.84 ± 2.49 vs. −19.7 ± 3.29, p = 0.244). No correlation was found between GLS and disease control (shown in **Figure 1**). Additionally, we did not find any statistical correlation between GLS values and disease duration or arterial hypertension in the acromegaly group (**Table 3**). The global circumferential strain and twist values were also similar between the two groups (−16.74 ± 3.18 vs. −16.6 ± 3.42, p = 0.909 and 14.55 ± 5.02 vs. 15.1 ± 3.94, p = 0.609, respectively). The global radial strain (GRS) values were lower in the acromegaly group than control group (36.88 ± 9.24 vs. 45.9 ± 14.2, p = 0.003). Measures of myocardial strain in both groups are summarized in **Table 4**.

**Table 3:** Correlation between GLS values and disease duration or arterial hypertension in the acromegaly group.

	GLS < 18% n = 9	GLS > 18% n = 16	p
Disease duration (years)			
<5	0 (0)	2 (12.50)	0.82
5-10	2 (22.22)	4 (25)	
>10	7 (77.78)	10 (62.5)	
Arterial Hypertension			
Yes	4 (44.44)	9 (56.25)	0.69
No	5 (55.56)	7 (43.75)	
Controlled disease			
Yes	1 (11.11)	6 (37.50)	0.36
No	8 (88.89)	10 (62.50)	

GLS, global longitudinal strain.

**Table 4:** Measures of myocardial strain in patients with acromegaly vs. control group.

	Acromegaly Group	Control Group	p
GLS	-18.84 ± 2.49	-19.7 ± 3.29	0.244
GCS	-16.74 ± 3.18	-16.6 ± 3.42	0.909
GRS	36.88 ± 9.24	45.9 ± 14.2	0.003
Twist	14.55 ± 5.02	15.1 ± 3.94	0.609

GLS, global longitudinal strain; GCS, global circumferential strain; GRS, global radial strain.

## Discussion

Cardiovascular disease is the most prevalent comorbidity in patients with acromegaly and has a direct relationship with the duration of the disease (26) and lack of hormonal control (5). However, progression to overt systolic dysfunction is uncommon, developing in <3% of patients (3).

Strain analysis is a modern, validated, and useful tool to detect subclinical systolic cardiac dysfunction in several clinical scenarios (12, 27–30). GLS has superior prognostic value compared with the traditional ejection fraction for predicting major adverse cardiac events (11). However, its use to provide earlier diagnosis of acromegalic cardiomyopathy has not been extensively studied to date. In the present study in which we compared a group of patients with long-term active acromegaly to control subjects, neither with cardiovascular symptoms, we found no significant differences in the various parameters of myocardial strain. To our knowledge, this is the first study to analyze all parameters of myocardial strain in this specific population.

We used STE to determine ventricular strain measurements in this study. We found no significant differences in GLS, circumferential strain, or ventricular twist when comparing patients with acromegaly and controls, even considering that the patients had an estimated disease duration of 11 years and that most patients (72%) had uncontrolled disease. We also found no significant difference in the mean LV GLS when comparing subgroups of patients with controlled and uncontrolled acromegaly.

We found a higher prevalence of diabetes mellitus in the acromegaly group than in the control group (64% vs. 25%). This may have been a confounding factor if the acromegaly group had exhibited compromised strain measurements because diabetes mellitus can lead to diabetic cardiomyopathy, which can decrease the GLS (9). However, despite the fact that the patients with acromegaly had this higher risk, we still did not find significantly lower GLS values in this group.

Our findings are consistent with those of Volscham et al. (14), who assessed myocardial strain values in 37 patients with active acromegaly compared with 48 controls and found no differences in GLS between the patients and controls. Likewise, they found no correlation between GLS and disease duration or IGF-1 level.

Conversely, Uziebło-Zyczkowska et al. (15) found impaired LV systolic function as assessed by GLS in 30 patients with acromegaly compared with 30 controls. This finding could have been due to the fact that although both groups had normal mean GLS values (−18.1% in the acromegaly group vs. −19.4% in the control group, p = 0.023), this difference was statistically significant.

Intriguingly, we found a reduction in GRS in the acromegaly group (36.88 ± 9.24 vs. 45.9 ± 14.2, p = 0.003). This is the first study to date to show this result in patients with acromegaly. However, although the absolute values of the GRS differed statistically from each other, they were within the normal range in both groups (between 35% and 59%) (31). Additionally, it is known that among the parameters of ventricular deformation, the GRS has the greatest number of limitations (31). Indeed, it has greater variability than the GLS (32), and such variability may be the result of a smaller portion of myocardial tissue needed to calculate GRS. Thus, small variations in the determination of the regions of interest could be associated with large differences in the result of radial strain (31).

The results of our study suggest that patients with acromegaly have a low risk of developing systolic dysfunction, and these findings are in line with more recent studies that show a trend toward lower cardiac complications in these patients (3) as well as a reduction in mortality by cardiac causes in the last decade (33). This could be a consequence of the better treatment of acromegaly and its cardiovascular comorbidities over time (34) or the difference between the pathophysiology of acromegaly-associated heart disease, which does not directly impair the cardiomyocyte contractility when compared with other causes of heart failure (35).

In fact, in the largest multicenter study designed to study the profile of patients with acromegaly, Petrossians et al. (36) found that only 1.6% of patients had heart failure. They also noted that patients with cardiac hypertrophy and heart failure at diagnosis were significantly older at diagnosis than those without cardiovascular complications (36). Therefore, they stated that because patients with acromegaly are living longer, the presence of acromegaly could simply represent an additional risk factor to many other contributory risk factors related to aging (36).

One of the limitations of the current study is its small sample size; however, it should be borne in mind that acromegaly is a rare disease. Additionally, this was a cross-sectional study, and a long-term prospective study could allow for a more detailed analysis of the evolution of functional cardiac parameters. Another potential limitation is the heterogeneity of the acromegaly group, since we had patients with disease duration ranging from 9 to 18 years and IGF-1 levels ranging from 1.04 to 2.54 times the upper limit of normal range. Additionally, SRL therapy has already been proved to be efficient in reducing ventricular hypertrophy (37) and in improving systolic and diastolic function (38). As a matter of fact, some studies have shown greater benefit of SRL therapy in reversing cardiomyopathy, when compared to surgical treatment alone, suggesting a possible direct beneficial of SRL on cardiomyocytes (39).

In conclusion, despite showing higher rates of LV hypertrophy, patients with acromegaly had no impairment of ventricular contractility as assessed by STE-derived myocardial strain when compared with a control group.

## Data Availability Statement

The raw data supporting the conclusions of this article will be made available by the authors, without undue reservation.

## Ethics Statement

The studies involving human participants were reviewed and approved by Human Research Ethics Committee of Hospital das Clínicas, Universidade Federal de Pernambuco. The patients/participants provided their written informed consent to participate in this study.

## Author Contributions

PG and LV designed the study, enrolled the patients and wrote the manuscript. ES performed all two-dimensional transthoracic echocardiographic studies and critically revised the manuscript. JC performed all strain analysis and contributed to the revision of the manuscript. All authors provided input and approved the final version.

## Conflict of Interest

The authors declare that the research was conducted in the absence of any commercial or financial relationships that could be construed as a potential conflict of interest.

## Publisher’s Note

All claims expressed in this article are solely those of the authors and do not necessarily represent those of their affiliated organizations, or those of the publisher, the editors and the reviewers. Any product that may be evaluated in this article, or claim that may be made by its manufacturer, is not guaranteed or endorsed by the publisher.
